# Nest boxes do not cause a shift in bat community composition in an urbanised landscape

**DOI:** 10.1038/s41598-020-63003-w

**Published:** 2020-04-10

**Authors:** Stephen R. Griffiths, Linda F. Lumsden, Kylie A. Robert, Pia E. Lentini

**Affiliations:** 1 Department of Ecology, Environment and Evolution, La Trobe University, Bundoora, 3086 Victoria Australia; 2 Arthur Rylah Institute for Environmental Research, Department of Environment, Land, Water and Planning, Heidelberg, 3084 Victoria Australia; 3 School of BioSciences, The University of Melbourne, Parkville, 3010 Victoria Australia

**Keywords:** Ecology, Conservation biology, Restoration ecology, Urban ecology

## Abstract

Nest boxes are often used to provide supplementary roosts for cavity-dependent wildlife, but little is known about if they influence faunal community composition. Long-term monitoring of bat boxes in south-eastern Australia indicated that their use was dominated by one generalist species (*Chalinolobus gouldii*), causing concern that installing bat boxes could cause a shift toward less diverse bat communities. To test this, we conducted a large-scale before-after control-impact experiment at 18 sites, over five years. Sites were either: (1) those with existing bat boxes, (2) those where boxes were added during the study, or (3) controls without boxes. We used echolocation call data from 9035 bat detector nights to compare community composition, diversity, and species’ relative activity between the sites. *Chalinolobus gouldii* continued to dominate the use of existing boxes, but we found little difference in community composition between sites based on the presence, absence, or addition of boxes. Our study is the first to explore the influence installing artificial hollows has on localized faunal assemblages over spatio-temporal scales relevant to management. We conclude that there is cause for optimism that bat boxes might not have perverse outcomes on local community composition in the short- to medium-term, as we had feared.

## Introduction

Tree hollows and cavities are keystone habitats used by a broad range of fauna for shelter and breeding^1^. The clearing of large, old trees during timber harvesting, urban developments, and for risk mitigation therefore presents a major ongoing threat for cavity-dependent wildlife^2^. Consequently, artificial cavities (nest or roost boxes) are often used to provide supplementary microhabitats for wildlife in human-disturbed landscapes where natural tree cavities have been depleted^3^. The installation of nest boxes has led to positive conservation outcomes for some species, such as the common hoopoe (*Upupa epops*)^4^, Gouldian finch (*Erythrura gouldiae*)^5^, and Leadbeater’s possum (*Gymnobelideus leadbeateri*)^6^. In these species-specific programs, boxes are designed in a targeted manner to attract the species of interest, whilst also attempting to exclude non-target taxa^7^. These programs also often adopt on-going systematic monitoring to empirically assess the effectiveness of a range of factors relating to the design and installation of the boxes, to increase their suitability for the target species^6,8^.

While a range of different box sizes, shapes and construction materials have been trialled for tree-cavity roosting insectivorous bats (hereafter ‘bats’), bat box designs are typically not species-specific^9^. They can be constructed from a range of materials, including timber^10^, plywood^11^, polyester resin^12^, and woodcrete^13^, and can be used by any species small enough to pass through the open slit entrance at the bottom of the box^10,11^. In spite of this, the majority of studies show that bat boxes are typically used by 1–3 mostly widespread and common species^9^, regardless of the local bat community assemblage. This is a concern for bat box programs in urbanised areas, where boxes are frequently deployed by land managers (e.g. local councils) and community groups targeting the whole bat community, not just individual species^14–16^.

Bats are often a relatively diverse and abundant component of the native mammalian fauna in urbanised landscapes^17,18^, but these communities are typically dominated by disturbance-adapted, generalist species^19,20^. These species are also more likely to roost in artificial structures than those with more specialised roosting requirements^19^. Hence, there is growing concern that the installation of bat boxes in urbanised areas may boost populations of widespread, disturbance-adapted species, to the detriment of less common, disturbance-sensitive species^9,16,21,22^.

Here, we build on key findings from a long-term bat-box monitoring program in Melbourne, south-eastern Australia. Bat boxes were installed by land managers and conservation-focused community groups at three suburban parks and one peri-urban park between 1994 and 2005, and monitoring has been regularly undertaken since. The aim of the bat box program was to provide supplementary artificial roosts for the community of bats present across Greater Melbourne^23,24^, which includes at least ten Vespertilionidae, three Molossidae, and one Miniopteridae^25^. However, one disturbance-adapted species with a generalist roosting ecology, the Gould’s wattled bat (*Chalinolobus gouldii*), has dominated the use of boxes at all four sites^16^. Annual trapping surveys conducted over more than 30 years at one site have also shown that the use of boxes by *C. gouldii* has corresponded to a disproportionate increase in its relative abundance in the local area^26^, though the generality of this finding warrants further investigation.

In this study, we conduct a large-scale, before-after control-impact (BACI) experiment to determine whether the addition of bat boxes leads to localised changes in bat community composition. We used ultrasonic bat detectors to document species’ relative activity while the bats are in flight at parks and reserves located within the urban matrix across Greater Melbourne, that either had existing bat boxes, had new bat boxes added during the study, or acted as control sites without bat boxes. We hypothesised that installing new bat boxes would cause localised changes in composition, such that communities would become less diverse and be dominated by widespread, disturbance-adapted species^16,19,21,26^. We discuss our findings in the context of empirically assessing the value of bat box programs for conserving entire bat communities in human-disturbed landscapes.

## Methods

Our study sites were 18 parks and reserves widely spread across the Greater Metropolitan area of Melbourne, Victoria, south-eastern Australia (37°48′S, 144°55′E, Table 1). We employed a BACI study design, and assigned sites to one of three treatment groups: (1) those with existing bat boxes, that had established populations of bats using them (‘existing boxes’; n = 4), (2) those where we added new bat boxes during the study (‘box addition’; n = 4), and (3) those that acted as control sites without bat boxes (‘control’; n = 10) (Table 1).

**Table 1:** Study site treatments and bat-box monitoring survey effort.

Name of park/reserve, suburb	Site code	Site treatment	No. boxes	Box check period	No. box checks
Gresswell Nature Conservation Reserve, Macleod	GNCR	Existing boxes	29	2005–2018	64
La Trobe University Wildlife Sanctuary, Bundoora	LTUWS	Existing boxes	37	2009–2018	50
Organ Pipes National Park, Keilor North	OPNP	Existing boxes	40	2012–2018	41
Wilson Reserve, Ivanhoe	WR	Existing boxes	20	2011–2018	78
Shepperds Bush Park, Wantirna South	SB	Box addition	24	2017	1
Woodlands Historic Park, Greenvale	WHP	Box addition	24	2017–2018	2
Westerfolds Park, Templestowe	WP	Box addition	24	2017	1
Yellow Gum Park, Plenty	YGP	Box addition	24	2017	1
Bolin Bolin Billabong, Bulleen	BBB	Control	0	n/a	n/a
Brimbank Park, Keilor East	BP	Control	0	n/a	n/a
Currawong Bush Park, Doncaster East	CBP	Control	0	n/a	n/a
Grange Heathland Reserve, Clayton South	GHR	Control	0	n/a	n/a
Plenty Gorge Park, South Morang	PGP	Control	0	n/a	n/a
Tullamarine Airport Greybox Woodland, Melbourne Airport	TA	Control	0	n/a	n/a
The 100 Acres Reserve, Park Orchards	TOA	Control	0	n/a	n/a
Valley Reserve, Mount Waverley	VR	Control	0	n/a	n/a
Yarra Bend Park, Fairfield	YBP	Control	0	n/a	n/a
Yan Yean Reservoir, Yan Yean	YYR	Control	0	n/a	n/a

During a single ‘box check’, all the boxes at that site were checked on the same day for the presence of bats.

### Bat detector surveys

Bat echolocation calls (henceforth ‘passes’) were recorded at all 18 sites concurrently using ultrasonic bat detectors (Anabat SD1 and SD2, Titley Scientific, Queensland, Australia). A single detector was placed along a flyway (a walking path or unsealed road) at each site inside a weatherproof box secured to the trunk of a tree at a height of 5 m. Detector microphones were housed within a plastic spout and angled upward at 45°, to prevent rain damage. All detectors were set to Division Ratio 8 and calibrated prior to deployment by adjusting their sensitivity levels against an ultrasound frequency generator^27^. They were programmed to start recording 1 h before sunset and to stop 30 min after sunrise, during which time the detectors were triggered automatically by ultrasonic noise.

We conducted ‘Before’ bat detector surveys at all 18 sites continuously over an 18-month period (4 September 2013 to 23 March 2015, henceforth the ‘Before–entire’ survey). During this period, the total number of survey nights per site ranged from 190–553; some detectors occasionally turned off when they could not get adequate charge from the solar panel due to cloud cover (Supplementary Material Table S1).

Ninety-six bat boxes constructed from marine plywood were installed across the four ‘box addition’ sites in April 2015 (Table 1). At each site, 24 boxes were attached to trees at heights ranging from 5–6 m above ground level: 16 single-chamber Bat Conservation International design boxes^11^, four cuboid-shaped boxes^28^, and four wedge-shaped boxes^29^ (Supplementary Material Fig. S1). A box check in December 2017 (32 months after installation) revealed that bats were using these new boxes, at which point a follow-up bat detector survey (i.e. post-impact) was conducted concurrently at all 18 sites. Detectors were placed at exactly the same locations at each site during the Before and After survey periods. The ‘After–autumn’ survey was carried out over 60 consecutive nights from 26 February to 26 April 2018. Because bat activity can vary seasonally according to species^30^, we took a subset of the Before–entire dataset that matched the exact dates of the After–autumn survey, for the sake of comparing community composition just within the autumn months (i.e. 26 February to 26 April 2014 and 26 February to 23 March 2015). This dataset is called the ‘Before–autumn’ survey herein.

We conducted bat detector surveys over much longer time frames than are typically employed for ecological studies investigating temporal patterns of bat activity. The extended survey length was intended to minimise the influence that variation in weather conditions can have on bat activity, both within and between nights^31–34^, and to maximise the precision of our estimates of different species’ relative activity^35,36^.

### Call and data analyses

For each study period and at each site we quantified community composition by calculating the relative activity of each species (number of passes per night per species). Bat passes were identified with automated AnaScheme software and a regionally specific identification key^37–39^. Several congeneric species present in the region cannot be reliably distinguished acoustically and so were combined into species complexes: *Nyctophilus* spp. includes both Gould’s long-eared bat (*Nyctophilus gouldi*) and lesser long-eared bat (*Nyctophilus geoffroyi*); and *Scotorepens* spp. includes the inland broad-nosed bat (*Scotorepens balstoni*) and eastern broad-nosed bat (*Scotorepens orion*)^25^. One obligate cave-roosting species, the eastern bent-winged bat (*Miniopterus orianae oceanensis*), occurs across parts of Melbourne^25,40^. Passes identified as *M. orianae oceanensis* were included in the analyses of bat community composition, however this species is not expected to use bat boxes. All other species are tree-cavity roosting bats and hence potentially could use the bat boxes. Bat passes that were very short, poor quality, or could not be identified to an individual species or a species complex were grouped into ‘unknown’ bats.

To visualise differences in bat community composition between the different treatments, we performed a non-metric multi-dimensional scaling (NMDS) ordination using the metaMDS function of the ‘vegan v2.5-2’ package^41^ in R 3.4.1^42^. The NMDS was performed across two dimensions on the site-by-species matrix using Bray-Curtis dissimilarity, where the species measure was the average number of passes per night per species across the respective survey periods. We produced two NMDS ordinations: the first was based on the Before–autumn data and was a comparison of the four sites with existing boxes and the 14 without, the second drew on the After–autumn data and was a comparison of the four sites with existing boxes, the four sites that had boxes added, and the 10 control sites. For both of these periods we conducted an ANOVA analysis based on Bray-Curtis dissimilarity using the ‘adonis2’ function and 999 permutations, to determine whether there were statistically significant differences between the treatment groups in the Before–autumn and After–autumn periods.

As a final step we wanted to determine whether there was an overall effect of the treatments on local bat community diversity. We calculated the Shannon-Wiener (H) diversity at each site, each night in the Before-autumn and After-autumn periods, based on the number of passes identified for each species using the ‘diversity’ function in the ‘vegan’ package. We then constructed a linear mixed-effect model (LMM) using the ‘lme’ function in the ‘nlme’ package in R, using this nightly diversity as our response variable. We fitted the survey period (Before–autumn or After–autumn) and site treatment (existing boxes, box addition, and control) as fixed effects, and included an interaction term between the two, specifically to test whether the addition of boxes caused a decrease in diversity in the After–autumn period. We also fitted the site as having a random effect on the intercept, and used a corARMA correlation structure (*p* = 1, *q* = 1) to account for temporal correlation between records taken from successive days.

### Long-term bat box checks

The four existing box sites are part of a long-term monitoring program, incorporating 126 bat boxes (Table 1). These boxes comprised nine designs based on those typically used in the Northern Hemisphere^11,28^, attached to trees at heights ranging from 4–6 m above ground level [see ^16^]. Boxes were checked during the day for the presence of bats at varying frequencies between 2005 and 2018 (Table 1). During the checks, all bats found roosting in boxes were collected and a range of biometric data were recorded for each individual. As part of an ongoing mark-recapture study, bats were also permanently marked with either a metal-alloy bat-band (Australasian Bird and Bat Banding Scheme) or microchip (Trovan ID100 Passive Implantable Transponder), enabling the total number of individuals to be determined on each check. All bats were either placed back in boxes on the same day (e.g. lactating females and their dependent young during the breeding season), or hand-released near the boxes after sunset.

### Ethical approval

All animal capture and handling procedures were carried out under ethics approval from the La Trobe University Animal Ethics Committee (Project Number AEC13-30). All experimental methods were carried out in accordance with relevant guidelines and regulations prescribed by the Department of Environment, Land, Water and Planning (Research Permit Number 10006790).

## Results

### Bat detector surveys

We recorded 2 938 721 bat passes over the combined 18-month Before–entire and the 60-day After–autumn surveys (from 9 035 detector nights across all sites combined), of which 1 074 262 passes (36.6%) were identified to 12 species or complexes. The bat community included the eight cavity-roosting species that used the boxes, plus the eastern false pipistrelle (*Falsistrellus tasmaniensis*), southern free-tailed bat (*Ozimops planiceps*), *Nyctophilus* spp., and the cave-roosting *M. orianae oceanensis* (Table 2). All 12 species or complexes were recorded at every site (Supplementary Material Fig. S2).

**Table 2:** The number of bat passes identified to species or species complexes during three survey periods: Before–entire (4 September 2013 to 23 March 2015); Before–autumn (26 February to 26 April 2014 and 26 February to 23 March 2015); After–autumn (26 February to 26 April 2018).

Species	Code	Before–entire	Before–autumn	After–autumn
**Vespertilionidae**				
*Chalinolobus gouldii*	Cg	288 851 (30.4%)	37 044 (23.2%)	21 100 (16.9%)
*Chalinolobus morio*	Cm	132 304 (13.9%)	23 385 (14.6%)	21 700 (17.4%)
*Falsistrellus tasmaniensis*	Ft	202 (0.02%)	28 (0.02%)	97 (0.08%)
*Nyctophilus* spp.	Nyct	43 752 (4.6%)	10 414 (6.5%)	6 129 (4.9%)
*Scotorepens* spp.	Scot	8 573 (0.9%)	1 589 (1.0%)	961 (0.8%)
*Vespadelus darlingtoni*	Vd	86 239 (9.1%)	17 280 (10.8%)	17 095 (13.7%)
*Vespadelus regulus*	Vr	21 540 (2.3%)	6 170 (3.9%)	3 677 (2.9%)
*Vespadelus vulturnus*	Vv	207 568 (21.9%)	35 468 (22.2%)	29 951 (24.0%)
**Molossidae**				
*Austronomus australis*	Aa	15 955 (1.7%)	3 967 (2.5%)	1 757 (1.4%)
*Ozimops planicep*s	Op	116 837 (12.3%)	21 005 (13.2%)	18 661 (15.0%)
*Ozimops ridei*	Or	1 196 (0.1%)	279 (0.2%)	416 (0.3%)
**Miniopteridae**				
*Miniopterus orianae oceanensis*	Moo	26 447 (2.8%)	3 012 (1.9%)	3 254 (2.6%)
**Summary**				
Total no. of passes		2 680 780	382 499	257 941
No. of passes identified		949 464	159 641	124 798
No. of passes unidentified		1 731 316	222 858	133 143

Numbers in parentheses are the percentage of identified passes for each taxa from the total number of identified passes during that survey period. Note that the Before–autumn survey is a subset of the 18-month Before–entire survey dataset.

*Chalinolobus gouldii* comprised the greatest proportion of activity across all 18 sites combined (28.9% of all identified passes), followed by *V. vulturnus* (22.1%), *C. morio* (14.3%), *O. planiceps* (12.6%), *V. darlingtoni* (9.6%) and *Nyctophilus* spp. (4.6%; Table 2). The remaining six taxa combined comprised <8% of all passes.

There was considerable variation in the species that accounted for the greatest proportion of detections at individual sites. *Chalinolobus gouldii* and *V. vulturnus* were the most recorded taxa at seven sites each (*C. gouldii*: two existing box, two box addition and three control sites; *V. vulturnus*: two existing boxes and five control sites). *Vespadelus darlingtonii* comprised the greatest proportion of detections at one box addition site and one control site, as did *C. morio* (Fig. S2).

Mean (±SD) daily maximum and minimum temperatures were similar during the Before–autumn (max 23.9 ± 4.9 °C, min 14.1 ± 2.9 °C) and After–autumn surveys (max 25.0 ± 4.6, min 14.0 ± 2.8 °C), and were comparable to Melbourne’s long-term averages over the same dates (26 February to 26 April 1964–2018, max 23.5 ± 4.5 °C, min 13.7 ± 2.9 °C). In contrast, there was some variation in precipitation across the survey periods. During the Before–autumn survey, there was a total of 76.6 mm of rainfall in 2014 and 49.4 mm in 2015, while 43.6 mm was recorded during the 2018 After–autumn survey. These values were all less than Melbourne’s long-term average total rainfall over the same dates (26 February to 26 April 1964–2018, 80.1 mm)^43^.

During the Before–autumn survey, 159 641 passes were identified to 12 taxa, with *C. gouldii* being the most detected species (23.2% of all passes), followed by *V. vulturnus* (22.2%; Table 2). During the After–autumn survey (26 February – 26 April 2018), 124 798 passes were identified to the same 12 taxa. *Vespadelus vulturnus* was detected at a similar proportion between the two periods (24.0%), while there were proportionally fewer *C. gouldii* detections during the After-autumn survey (16.9%; Table 2).

**Figure 1:**
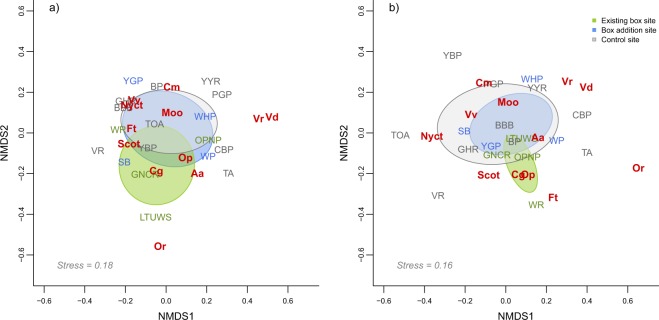
NMDS ordinations of bat community composition, based on the average number of passes of each species per night during two survey periods: (**a**) Before–autumn, 26 February to 26 April 2014 and 2015; and (**b**) After–autumn, 26 February to 26 April 2018. Sites are grouped according to treatment; species codes are in red. For full site names and species codes see Tables 1 and 2.

**Figure 2:**
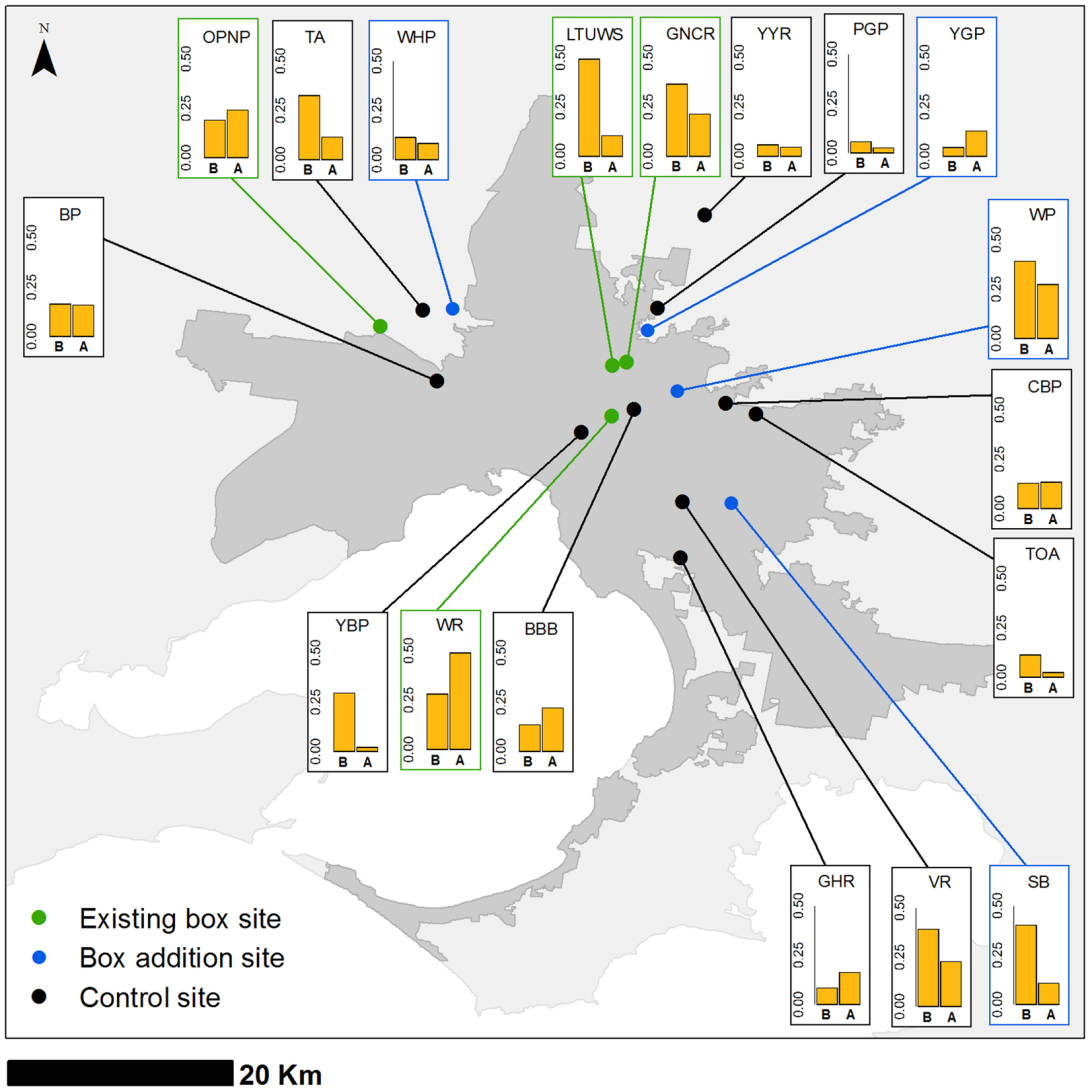
Locations of the 18 sites grouped by three treatments (existing boxes, box addition, and control) across Greater Melbourne (dark grey shaded area), Victoria, Australia. Bar charts show proportion of echolocation passes identified as *Chalinolobus gouldii* at each site during the (B) Before–autumn and (A) After–autumn surveys. For full site names and species codes see Tables 1 and 2. This map was constructed in R 3.4.1^42^ and ArcMap v. 10.7^67^, using spatial data that were obtained from open access sources^68,69^.

**Figure 3:**
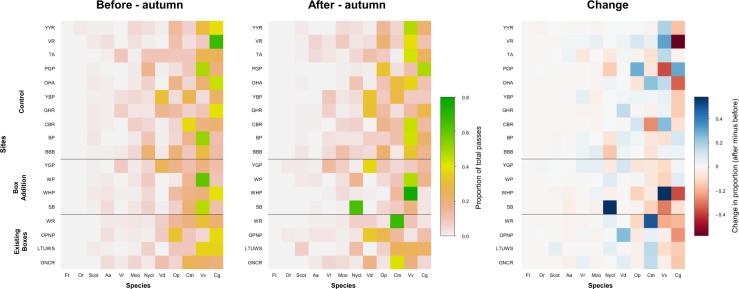
Site by species matrices, shaded according to (**a**) the proportion of total passes made up by each species in the ‘Before–autumn’ period, and (**b**) the proportion of total passes in the ‘After–autumn’ period. Because these are proportions, rows sum to 1. In these plots the species are arranged from the least to most dominant, left to right, and the sites are arranged by treatment groups (existing boxes, box addition, control), with different groups being separated by the horizontal black lines. For full site names and species codes see Tables 1 and 2. Plot (**c**) represents the difference between (**a**,**b**) (i.e. is the ‘After–autumn’ proportion minus the ‘Before–autumn’ proportions) – redder colours indicate that the species became less dominant at that site in the ‘After–autumn’ period, and bluer colours indicate that it became more dominant. If the addition of boxes caused the most common species to become more dominant in the community (and hence make up a greater proportion of passes) then we would expect there to be bluer colours in the far right of the ‘Addition’ cells in (**c**), but with the exception of *Vespadelus vulturnus* (Vv) becoming more dominant at the Woodlands Historic Park (WHP) site, the colours are mostly at the redder end of the spectrum.

The NMDS ordination indicated that there was little difference in community composition between the site treatments during the Before–autumn and After–autumn surveys (Fig. 1). This was further supported by the subsequent ANOVA, which showed that the bat community did not differ between site treatments during either the Before–autumn (boxes vs no boxes, F = 0.92, d.f. = 1, *P* = 0.49) or After–autumn periods (existing boxes vs boxes added vs control, F = 1.33, d.f. = 2, *P* = 0.21). There was some evidence of *C. gouldii* comprising a greater proportion of overall bat activity at three of the existing box sites during the Before–autumn survey (Figs. 2, 3a, and Supplementary Material Fig. S2). However, during the After–autumn survey, this species was detected less often and *V. vulturnus* had the highest activity levels at these sites (Figs. 2, 3b and S2). Similarly, at three of the box addition sites, the proportion of *C. gouldii* was lower during the After–autumn survey than the Before–autumn survey (Figs. 2, 3 and S2). Across the ten control sites there were no consistent patterns in the relative activity of *C. gouldii* from the Before–autumn to After–autumn detector surveys (Figs. 2, 3 and S2).

**Figure 4:**
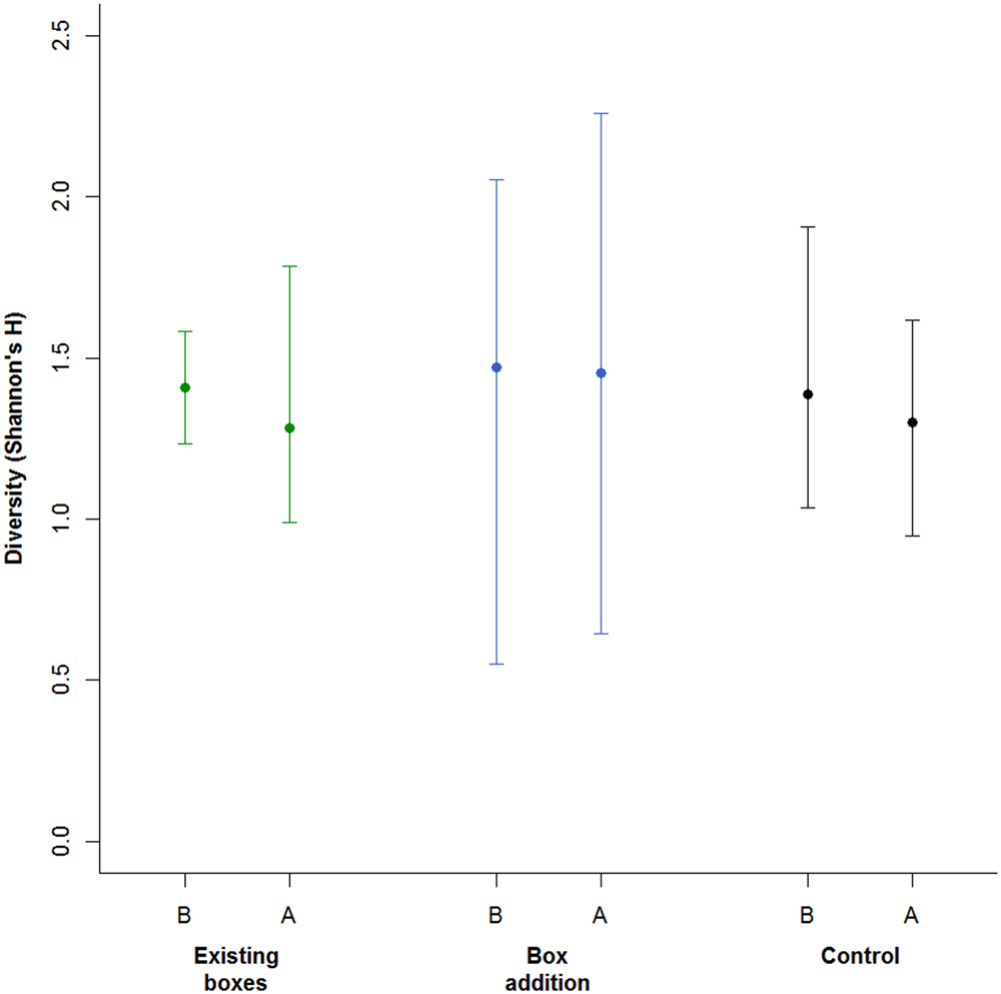
Estimated Shannon-Wiener (H) diversity during the Before–autumn (B) and After–autumn (A) survey periods, based on the number of passes identified for each species per night at each site. Sites are grouped according to treatment (existing boxes, box addition, control). Error bars represent 95% confidence intervals.

Based on the LMM of nightly Shannon-Wiener diversity, there was no evidence that there was a shift in local bat community diversity in response to the addition of the bat boxes (Fig. 4, Supplementary Material Table S2). If anything, the estimated nightly diversity in the After–autumn period was slightly higher at the box addition sites (1.45, cf 1.28 and 1.30 for the existing boxes and control sites, respectively), and there was less of a decline in diversity relative to the Before–autumn period (decline of 0.019 at the addition sites cf 0.12 for the existing boxes and 0.087 for the control sites), though there was also substantial error around those estimates (Fig. 4, Table S2).

### Long-term bat box checks

A total of 5 491 individually banded or microchipped bats, comprising eight species, were recorded using boxes across the four existing box sites between 2005 and 2018. *Chalinolobus gouldii* was the most common species found using boxes (2 703 females and 2 201 males, 89.3% of the total numbers), both across all the sites and at each individual site, and was the only species that used the boxes as maternity roosts over multiple years at all sites (Table 3; Supplementary Material Fig. S3).

**Table 3:** The number of banded or microchipped bats that used boxes at the four existing box sites from 2005–2018.

Species	Site code				Total (percentage)
	GNCR	LTUWS	OPNP	WR	
**Vespertilionidae**					
*Chalinolobus gouldii*	1 436	1 389	1 530	549	4 904 (89.3%)
*Chalinolobus morio*	8	1	6	19	34 (0.6%)
*Scotorepens orion*	0	1	0	39	40 (0.7%)
*Vespadelus darlingtoni*	9	3	128	18	158 (2.8%)
*Vespadelus regulus*	0	0	1	0	1 (0.02%)
*Vespadelus vulturnus*	0	0	9	0	9 (0.2%)
**Molossidae**					
*Austronomus australis*	149	49	141	5	344 (6.3%)
*Ozimops ridei*	0	0	1	0	1 (0.02%)
Total	1 602	1 443	1 816	630	5 491

For full site names and site-specific survey effort see Table 1. Numbers in parentheses are the percentage of each species of the total number of marked bats across the four sites.z.

**Figure 5:**
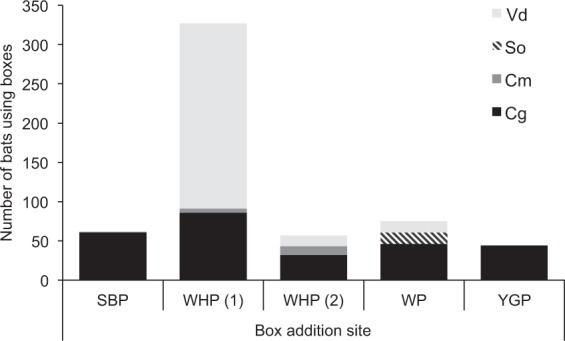
Summary of bats using boxes during checks conducted in December 2017 at the four box addition sites. For full site names and species codes see Tables 1 and 2. Data from the two checks at WHP are shown separately: (1) 13 December 2017, and (2) 24 May 2018. Note – the *Scotorepens* spp. found using boxes was *S. orion*.

During the single check of the four box addition sites in December 2017, a total of 508 bats, comprising four species, were recorded using boxes. *Vespadelus darlingtoni* was the most common species (251 individuals), followed by *C. gouldii* (237 individuals), *S. orion* (15 individuals), and *C. morio* (5 individuals). Most of the *V. darlingtoni* (236) were from one site (WHP), while *C. gouldii* comprised the majority of records at the remaining three sites (Fig. 5).

Maternity groups comprising adult females and dependent young of three species (*C. gouldii*, *S. orion* and *V. darlingtoni*) were found using the new boxes. Across all four sites, 108 of the 237 *C. gouldii* were juveniles. *Vespadelus darlingtoni* bred at two sites, seven of 14 individuals at WP were juveniles, while 115 of the 236 individuals at WHP were juveniles (Fig. S3). *Scotorepens orion* was only recorded at one site (WP; Fig. 5), where five of the 15 individuals were juveniles.

As considerably more *V. darlingtoni* were recorded during the first check at WHP than have been recorded using boxes previously in Australia, a follow-up check was conducted at this site on 24 May 2018. During this check there were only 14 *V. darlingtoni* found roosting in the boxes, along with 32 *C. gouldii* and 11 *C. morio* (Fig. 5).

## Discussion

Nest boxes are a common means of providing supplementary microhabitats for cavity-dependent wildlife; however, little is known about their influence on faunal community composition^9,44^. Here, through manual bat box checks and long-term passive acoustic surveys, we compared community composition at four sites where boxes have been used by bats for more than a decade^16^ with sites that either did not have boxes, or sites where boxes were added as part of a BACI experiment. We had predicted that the relative activity of one generalist species (*C. gouldii*) would be greater, and that community diversity would decline, at sites where bat boxes were added compared to sites without boxes. However, despite the fact that *C. gouldii* continued to dominate use of the existing bat boxes, we found little difference in community composition across the 18 study sites between the ‘Before’ and ‘After’ study periods. The relative activity of each species or complex differed between the sites, with no clear or consistent patterns corresponding to the different treatments and survey periods. This suggests that, while use of bat boxes by widespread, adaptable species (such as *C. gouldii*) may have some influence on localised patterns of community composition^26^, it does not necessarily result in a consistent, widespread dominance of generalist species, or a reduction in overall bat diversity.

During this study, the factors driving patterns in bat community composition were likely to be acting over larger spatial scales than the parks where we conducted bat detector surveys. For example, studies have shown that various forms of disturbance associated with urbanisation, such as artificial night-time lighting^45–47^, housing density^40,48,49^, tree cover^50,51^, the position of major roads^52–54^, and distance to surface water and vegetation structure around waterways^25,55,56^, form complex interactions that drive spatiotemporal patterns of species distribution, abundance and relative activity. Our results suggest that some combination of the above-mentioned factors, along with variation in weather conditions^31^ and abundance of nocturnal arthropods^57^, were stronger drivers of community composition than the presence or absence of bat boxes.

Our long-term mark-recapture data showed that large, discrete populations of *C. gouldii* used boxes over repeated years at the four existing box sites. These populations included resident adults recaptured over multiple years, juveniles born into the population every year (some of whom subsequently migrated), and immigrants that entered the population. Nonetheless, it remains unclear whether localised increases in numbers of *C. gouldii* occurred at the existing box sites as a direct result of long-term box use^26^, or rather that there was a redistribution of the local populations as individuals incorporated bat boxes into their suite of roost sites, along with natural hollows^21^. While our large bat detector survey effort is likely to have produced accurate measures of the relative activity of different species^35,36^, it is not possible to determine whether the passes were made by members of local colonies roosting at sites with or without boxes, or by individuals that were roosting elsewhere, but were using the site for foraging or commuting.

The question of whether bats that used boxes were members of existing local populations, or individuals that immigrated into a site, is equally relevant to other species that used the newly installed boxes, such as *V. darlingtoni*. The large breeding population of *V. darlingtoni* found at WHP in December 2017 was surprising, especially as it was within 32 months of installing the boxes. As the numbers varied widely between the two box checks at this site, it may be that members of the local population of *V. darlingtoni* incorporated the new bat boxes into the suite of roosts that they use, which would have previously comprised only natural tree cavities or buildings. Box checks and detector surveys would need to be conducted over extended periods (e.g. 5–10 years) to effectively document changes in local population sizes and community structure, as the 32-month time period since box installation may not have been sufficient to reveal significant increases in breeding success leading to larger population numbers. Ultimately, surveys combining trapping, banding and radio-tracking of marked individuals before and after the addition of boxes may be required to determine the source of colonies of bats using boxes.

The vast number of nest boxes placed in the environment worldwide represents habitat supplementation on a similar scale to other well-intentioned human activities, such as bird feeding^58^. Interestingly, large-scale bird feeding has been shown to strongly influence the structuring of bird communities in urbanised areas^59^. It is therefore surprising that only limited research has addressed similar questions relating to the potential flow-on effects of nest box programs^60^. To our knowledge, our study is the first to explore the link between the provision of artificial cavities and changes in bat community composition over spatial and temporal scales relevant to management, and over time frames in which boxes become occupied. While we did not find strong evidence that bat box use influenced localised community structure, this is an area that warrants investigation for bat box programs conducted in other systems, and more broadly for nest box programs targeting other groups of cavity-dependent wildlife. Studies employing BACI surveys designed to monitor changes in localised community composition and species’ abundance in situations where nest boxes are used to compensate for the removal of mature, cavity-bearing trees^3^ would be particularly informative.

Our long-term bat box program has been successful in attracting several bat species, some of which use the boxes throughout the year, including as maternity roosts. The mark-recapture data generated has helped us gain insights into various aspects of the ecology of the bats that use the boxes^61,62^. While the program has not achieved the *a priori* objective of providing supplementary roosting habitats for all species of bats (i.e. at the community level), it does not appear to have had an overall detrimental effect on localised bat communities across Greater Melbourne, as we had predicted^16,26^. Our findings suggest that, despite large resident populations of one highly adaptable, generalist species consistently using boxes, that the parks and reserves that we sampled, and the surrounding areas across Melbourne’s highly modified urban landscape, have adequate foraging and roosting resources to maintain a relatively diverse community of cavity-roosting bats.

Given that many disturbance-sensitive species have not used bat boxes to date^9^, there is great need for developing and testing novel techniques of providing supplementary roosts for cavity-dependent bats in human-disturbed environments;^16,63,64^ for example, using chainsaws to mechanically excavate cavities into the trunk or branches of trees^65^. However, where possible, the retention of mature, hollow-bearing trees should be the primary objective of conservation and management programs targeting cavity-dependent wildlife in disturbed landscapes^2^, such as parks and reserves located within an urban matrix^66^.

## Supplementary information

Supplementary Information.

Supplementary Information 2.

## Data Availability

The datasets generated during and/or analysed during the current study are available from the corresponding author on request.
