# Supplementary material for: Nest boxes do not cause a shift in bat community composition in an urbanised landscape
Source: Sci Rep. 2020 Apr 10;10:6210. doi: 10.1038/s41598-020-63003-w (PMC7148353; doi:10.1038/s41598-020-63003-w)
Supplement: Supplementary file 1 — Supplementary Information. [file 41598_2020_63003_MOESM1_ESM.pdf]

Title: Nest boxes do not cause a shift in bat community composition in an urbanised landscape

Authors: Stephen R. Griffiths[1]*, Linda F. Lumsden[2], Kylie A. Robert[1], Pia E. Lentini[3]

[1]Department of Ecology, Environment and Evolution, La Trobe University, Bundoora Victoria, Australia 3086

[2]Arthur Rylah Institute for Environmental Research, Department of Environment, Land, Water and Planning, Heidelberg, Victoria, Australia 3084

[3]School of BioSciences, The University of Melbourne, Parkville, Victoria, Australia 3010

***Corresponding author:** s.griffiths@latrobe.edu.au

Journal: Scientific Reports

Supplementary Material – Tables

S1. Bat detector survey effort

Table S1. Bat detector nights from surveys at 18 sites grouped by three treatments: (1) existing boxes, (2) box addition, and (3) control. The 'Before–entire' survey was conducted from 4 September 2013 to 23 March 2015; the 'After–autumn' survey was from 26 February to 26 April 2018. For full site names see Table 1.

		**Before–entire**				**After–autumn**	**Total bat detector nights**
**Site treatment**	**Site code**	**Autumn**	**Spring**	**Summer**	**Winter**		
Existing boxes	GNCR	94	155	114	87	56	506
Existing boxes	LTUWS	56	159	167	63	56	501
Existing boxes	OPNP	115	166	180	92	51	604
Existing boxes	WR	105	137	106	92	58	498
Box addition	SB	93	142	180	27	55	497
Box addition	WHP	94	116	169	92	56	527
Box addition	WP	64	102	180	35	49	430
Box addition	YGP	96	76	106	92	59	429
Control	BBB	86	57	43	4	49	239
Control	BP	86	121	124	70	56	457
Control	CBP	87	138	180	34	56	495
Control	GHR	97	156	180	42	55	530
Control	PGP	104	172	180	92	56	604
Control	TA	109	159	180	92	56	596
Control	TOA	97	156	180	92	49	574
Control	VR	94	156	180	28	55	513
Control	YBP	81	96	180	47	55	459
Control	YYR	96	149	180	92	59	576
						**Total**	**9 035**

S2. Linear mixed-effects model estimates for nightly Shannon-Wiener diversity

Table S2. Parameter estimates for the linear mixed-effects model for the response of site-level diversity (Shannon's H) to the Time period (Before–autumn or After–autumn) and Treatment (Existing boxes, Box addition, and Control).

Fixed effect	Estimate	Standard error
Intercept (Time: Before–autumn, Treatment: Existing)	1.413	0.089
Time: After–autumn	-0.082	0.033
Treatment: Control	-0.059	0.105
Treatment: Box addition	-0.001	0.125
Time: After–autumn × Treatment: Box addition	-0.002	0.039
Time: After–autumn × Treatment: Box addition	0.14	0.046
