# Supplementary material for: Nest boxes do not cause a shift in bat community composition in an urbanised landscape
Source: Sci Rep. 2020 Apr 10;10:6210. doi: 10.1038/s41598-020-63003-w (PMC7148353; doi:10.1038/s41598-020-63003-w)

Title: Nest boxes do not cause a shift in bat community composition in an urbanised landscape

Authors: Stephen R. Griffiths[1]*, Linda F. Lumsden[2], Kylie A. Robert[1], Pia E. Lentini[3]

[1]Department of Ecology, Environment and Evolution, La Trobe University, Bundoora Victoria, Australia 3086

[2]Arthur Rylah Institute for Environmental Research, Department of Environment, Land, Water and Planning, Heidelberg, Victoria, Australia 3084

[3]School of BioSciences, The University of Melbourne, Parkville, Victoria, Australia 3010

***Corresponding author:** s.griffiths@latrobe.edu.au

Journal: Scientific Reports

Supplementary Material – Figures

Bat box designs

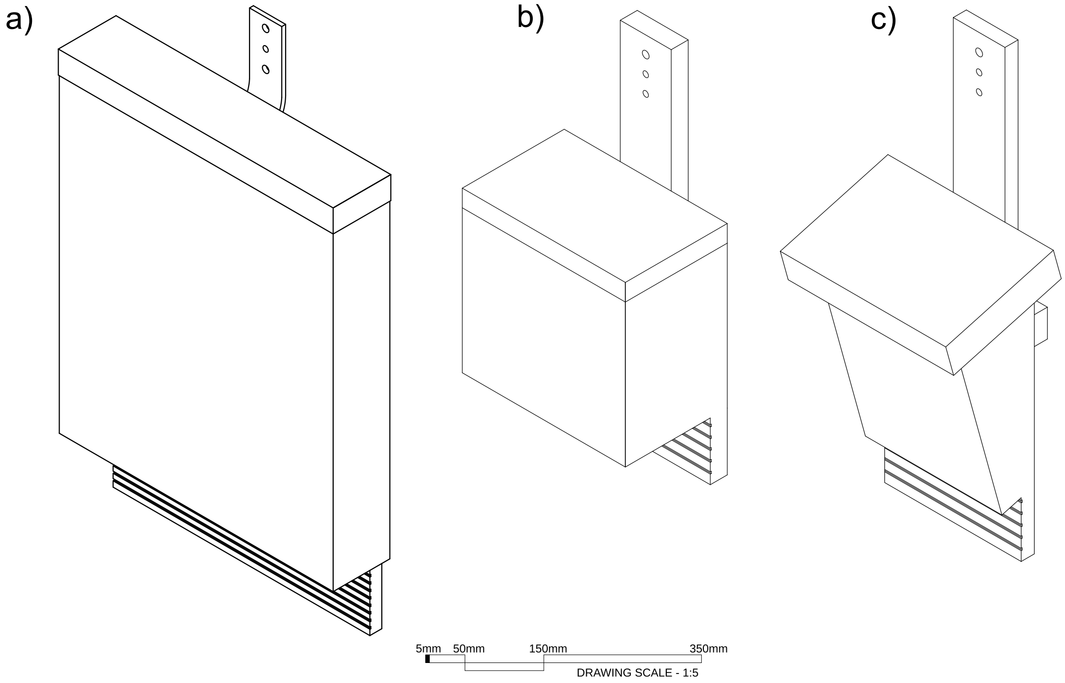

Fig. S1. Diagrams of the three different bat box designs that were installed at the four 'box addition' sites (see Table 1). (a) Single-chamber 'Bat Conservation International' design boxes (Tuttle et al., 2013), (b) cuboid-shaped boxes (Stebbings and Walsh, 1985), and (c) wedge-shaped boxes (Hines 1985).

Summary of identified echolocation calls

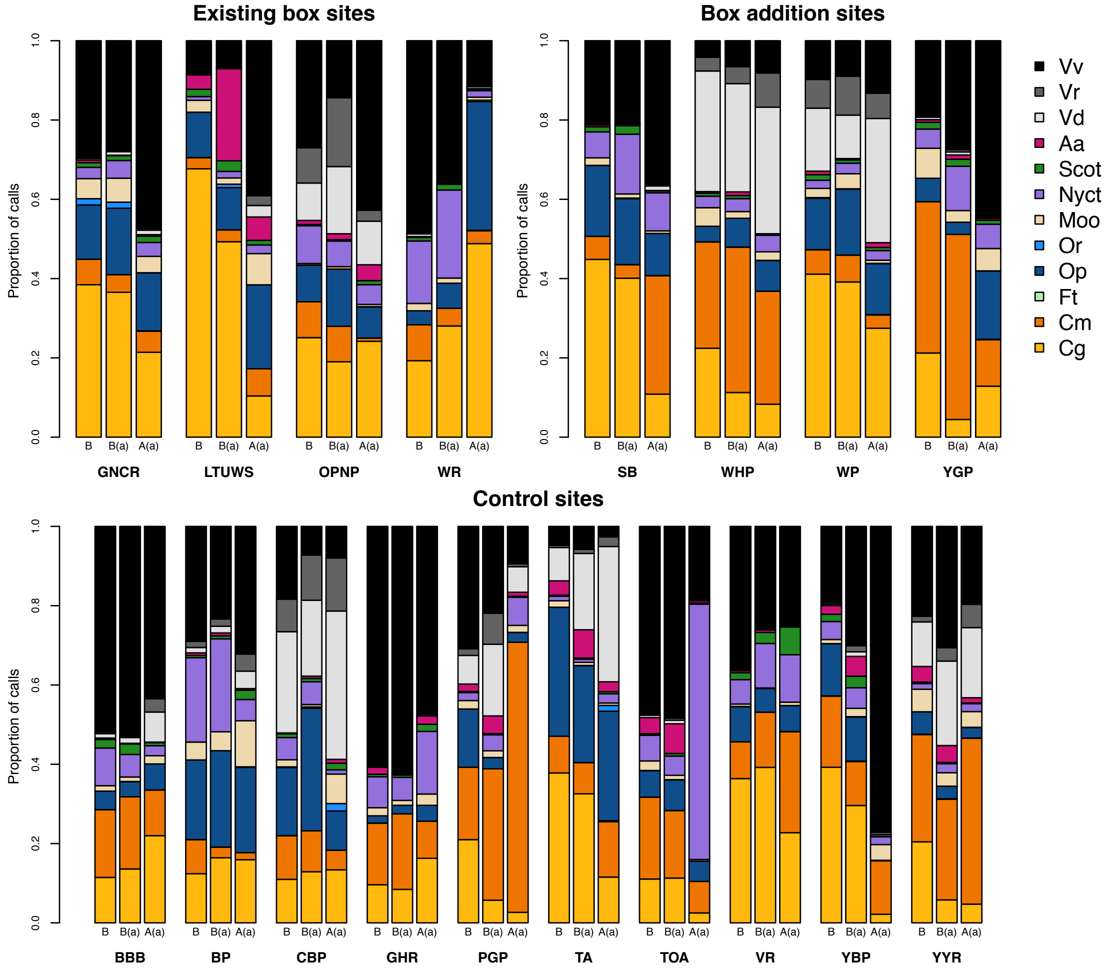

Fig. S2. The proportion of identified echolocation passes from bat detector surveys at 18 sites grouped by three treatments: (1) existing boxes, (2) box addition, and (3) control. For full site names and species codes see Tables 1 and 2. For each site, columns represent data from three survey periods: B = Before–entire, 18 months from 4 September 2013 to 23 March 2015; B(a) = Before–autumn, 26 February to 26 April 2014 and 2015; A(a) = After–autumn, 26 February to 26 April 2018.

Summary of long-term box use

Long-term mark-recapture data showed that the largest discrete population of *C. gouldii* was at OPNP, where 415.0 ± 96.3 individuals used the boxes annually. The next two most common species using boxes at OPNP had much smaller populations (*A. australis*, 32.3 ± 19.2 individuals per year; *V. darlingtoni*, 25.7 ± 12.9 individuals per year; Fig. S3c). The numbers of *C. gouldii* using boxes annually were comparable at GNCR (183.4 ± 51.9 individuals per year; Fig. S3a) and LTUWS (142.4 ± 109.3 individuals per year; Fig. S3b). The smallest number of *C. gouldii* using boxes annually was at WR (111.6 ± 32.4 individuals per year), however this still represented 22 times more *C. gouldii* than the next most common box-using species, *S. orion* (4.9 ± 2.3 individuals per year; Fig. S3d).

Chalinolobus gouldii was the only species that used the boxes as maternity roosts over multiple years at all sites. A total of 2 501 juvenile *C. gouldii* were recorded across the four existing box sites from 2005–2018. The mean (±SD) number of juvenile *C. gouldii* recorded each year ranged from 167.2 ± 59.8 at OPNP to 33.3 ± 11.6 at WR. The two largest annual breeding events recorded were 246 juvenile *C. gouldii* at OPNP in 2013, and 232 juveniles at LTUWS in 2017. Four other species also bred in boxes, but at much lower rates, and mostly only at a single site. For example, eight juvenile *V. darlingtoni* were recorded at OPNP, six juvenile *S. orion* at WR, and two juvenile *C. morio* at WR. A total of 28 juvenile *A. australis* were recorded across three sites: 20 individuals at GNCR, five at LTUWS, and three at OPNP (Fig. S3).

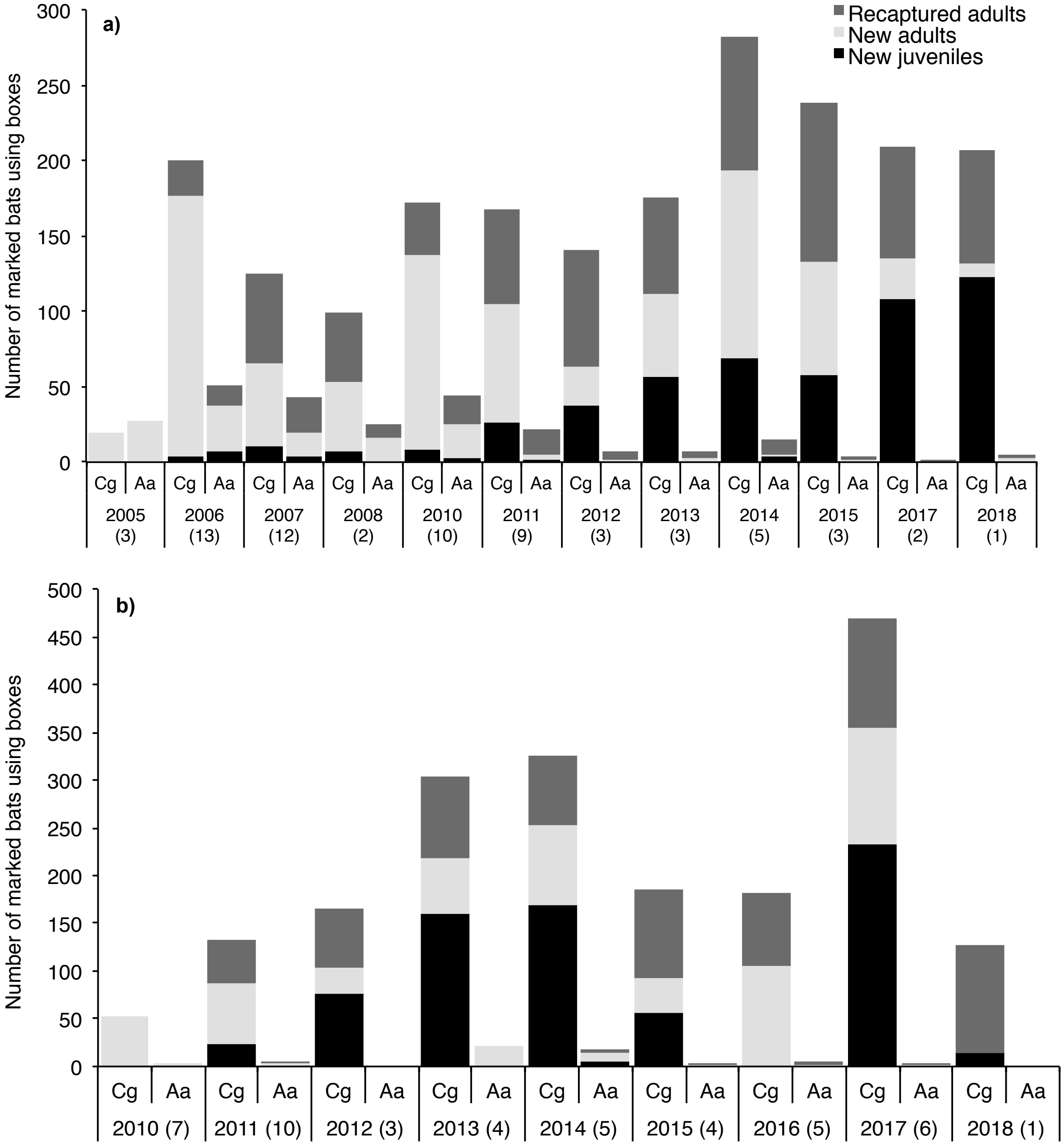

Fig. S3. Mark-recapture records of banded or microchipped bats using bat boxes at the four existing box sites in Melbourne, Australia: (a) GNCR, (b) LTUWS, (c) OPNP, and (d) WR. For full site names and species codes see Tables 1 and 2. Numbers in parentheses represent the total number of checks (when all boxes were checked on the same day) at that site in that year. For each site, only species that accounted for ≥ 5% of all marked bats are shown (see Table 2).

Fig. S3 – continued.

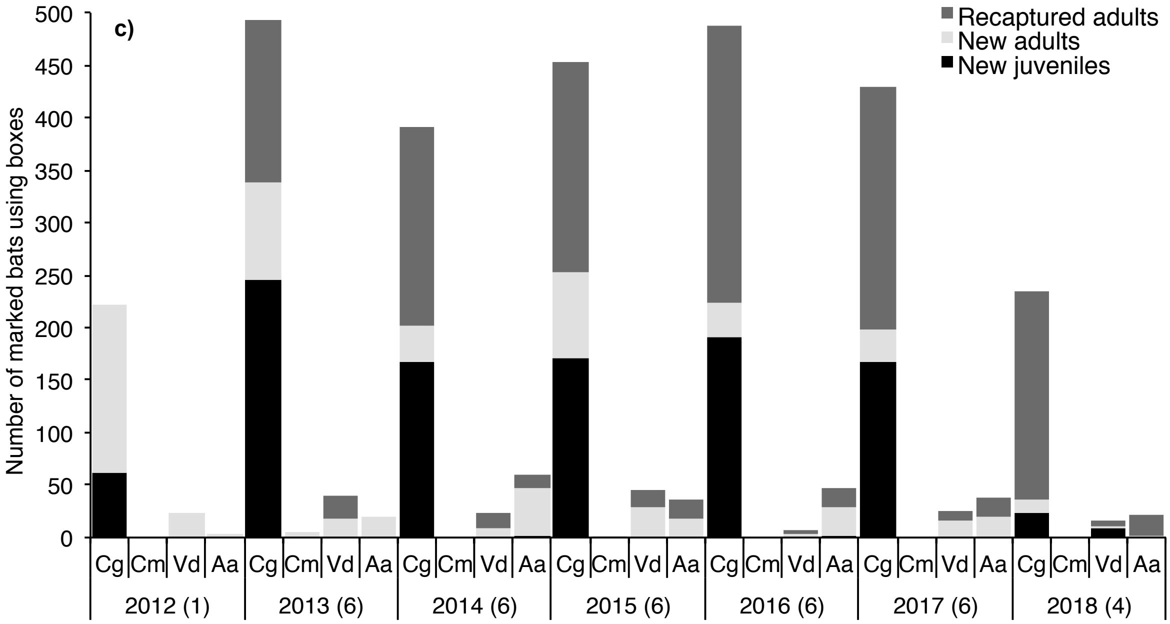

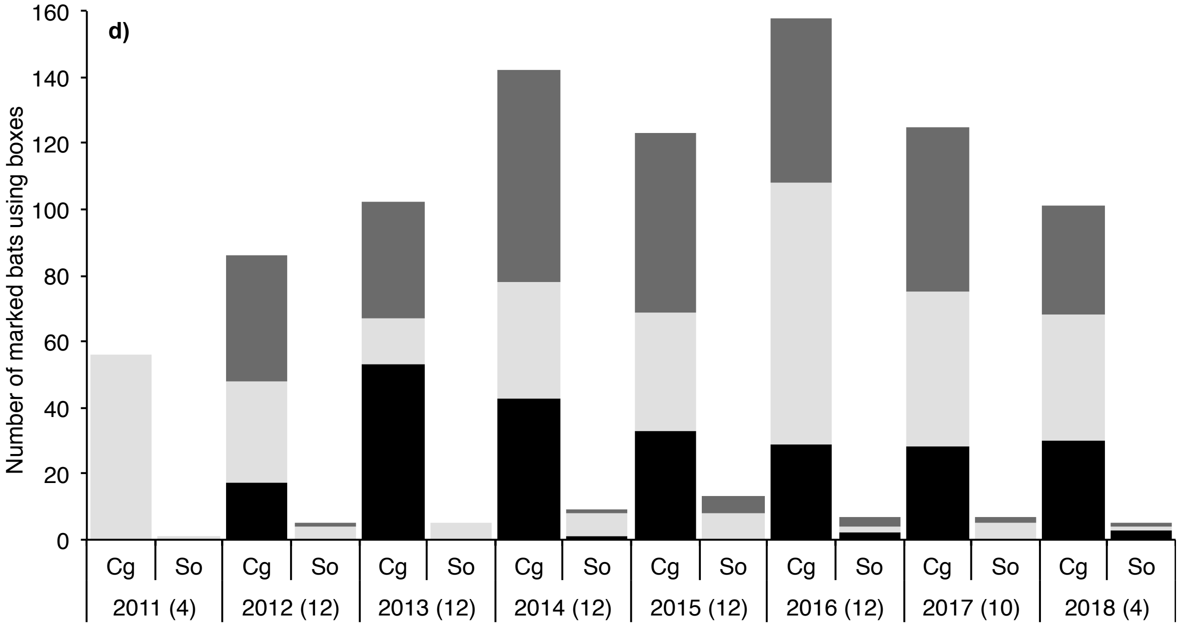

Supplement: Supplementary file 2 — Supplementary Information 2. [file 41598_2020_63003_MOESM2_ESM.pdf]
